# Rare Presentation of Gastric Myeloid Sarcoma

**DOI:** 10.14309/crj.0000000000001973

**Published:** 2026-01-20

**Authors:** Shailavi Jain, Austin Dickerson, Bhavani Moparty, Hemangi Kale

**Affiliations:** 1 Department of Gastroenterology, Baylor University Medical Center, Baylor Scott & White Health, Dallas, TX; 2 Department of Gastroenterology, Texas Digestive Disease Consultants, GI Alliance, Dallas, TX; 3 Department of Gastroenterology, Digestive Health Associates of Texas, GI Alliance, Dallas, TX

**Keywords:** myeloid sarcoma, acute myeloid leukemia, extramedullary, endoscopy

## CASE REPORT

Myeloid sarcomas, an extramedullary manifestation of myeloid conditions, rarely develop in the gastrointestinal tract.^1,2^ They are frequently associated with acute myeloid leukemia and often misdiagnosed.^3,4^

**Figure 1.**
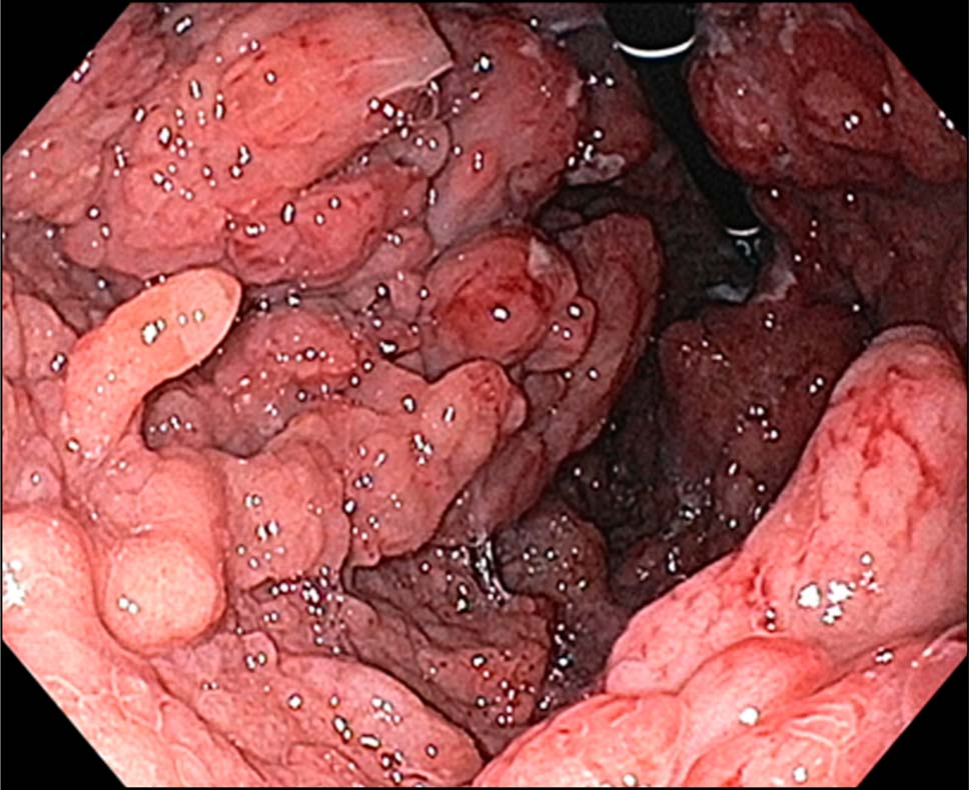
Esophagogastroduodenoscopy findings of polypoid gastric mucosa in the fundus.

**Figure 2.**
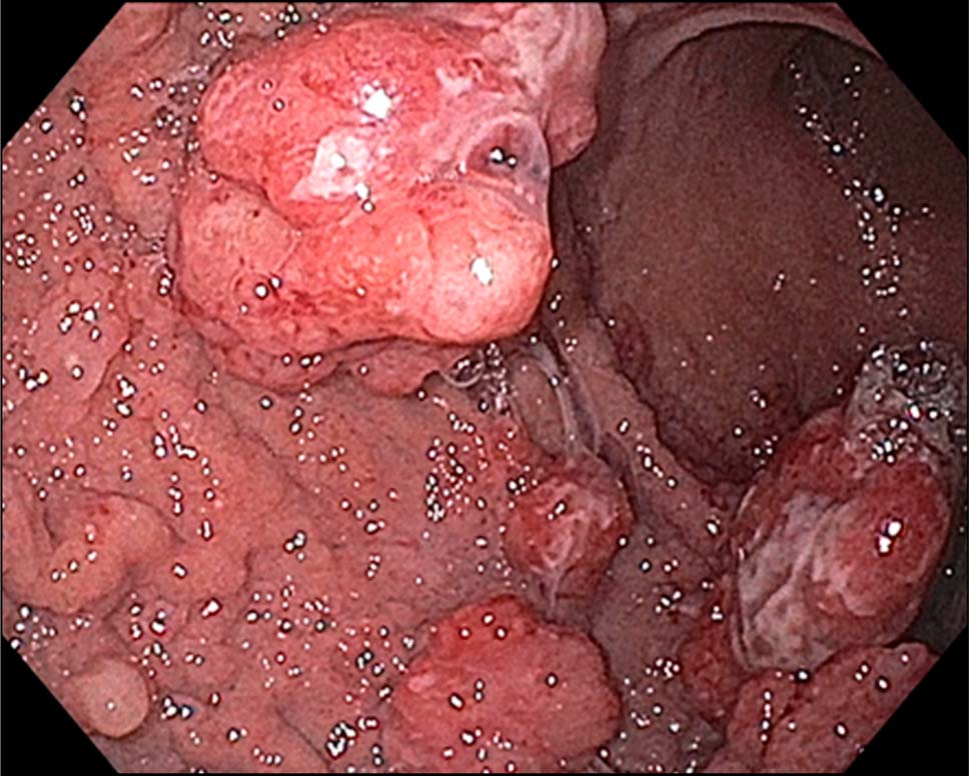
Esophagogastroduodenoscopy findings of polypoid gastric mucosa with antral sparing.

**Figure 3.**
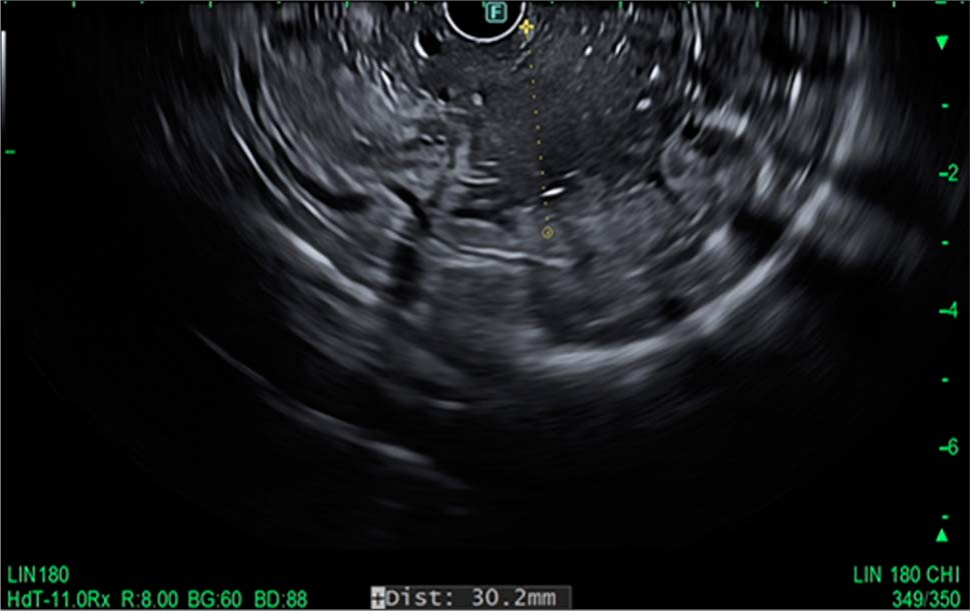
Endoscopic ultrasound findings of gastric wall thickening within the deep mucosa (layer 2).

**Figure 4.**
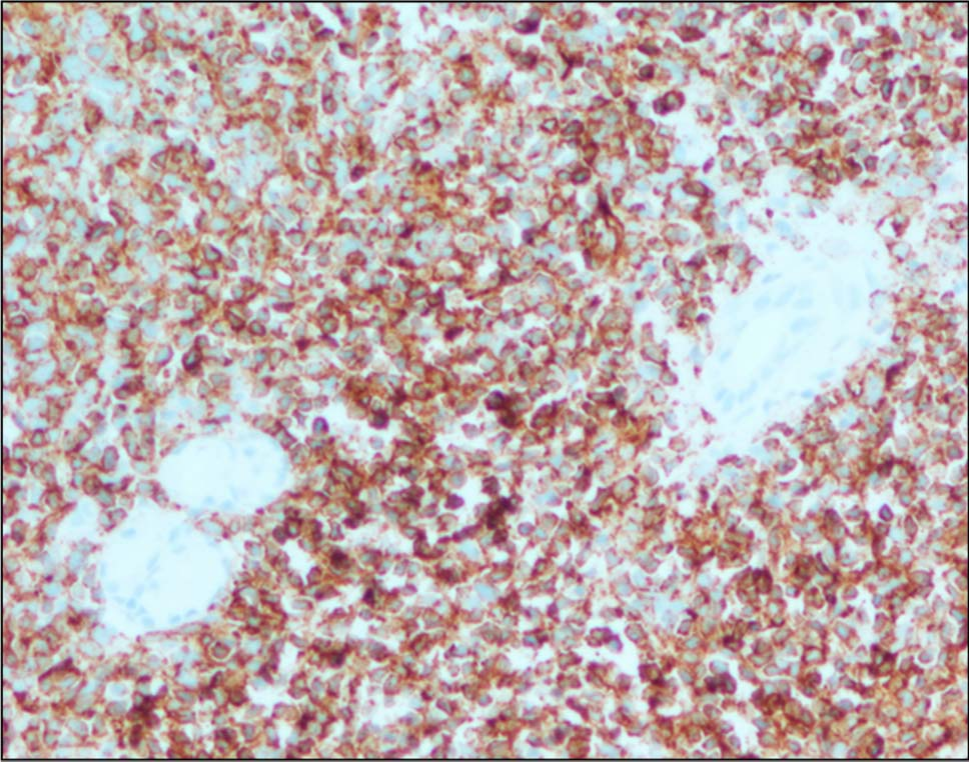
CD34 immunohistochemical staining highlights the atypical cellular infiltrate (200× magnification). The sample was also positive for terminal deoxynucleotidyl transferase, scattered positive for CD19 and lysozyme, and negative for CD10, CD117, CD3, CD79a, CD20, and myeloperoxidase.

A 43-year-old man with a history of relapsing acute myeloid leukemia and chronic sclerodermatous graft-versus-host disease on maintenance tacrolimus and ruxolitinib presented with abdominal pain and melena. Upper endoscopy and endoscopic ultrasound showed polypoid mucosa with friability and ulceration from the cardia to the body, with antral sparing (Figures 1 and 2) with gastric wall thickening within the deep mucosa up to 30 mm (Figure 3). Mucosal biopsies and fine needle aspiration were performed. Histology showed atypical immature cell proliferation which was positive for immature lymphoid/leukemic markers, CD34, and terminal deoxynucleotidyl transferase (Figure 4).

The patient was diagnosed with relapsed acute myeloid leukemia with extramedullary involvement. Bone marrow biopsy demonstrated increased blasts with hypocellular marrow, confirming the diagnosis. He started reinduction chemotherapy with high-dose cytarabine and intrathecal methotrexate and had partial response; however, he succumbed to neutropenic septic shock.

The presence of a myeloid sarcoma portends a worse prognosis in acute leukemia and is typically more refractory to treatment.^5^ This entity should be recognized by gastroenterologists to allow for prompt diagnosis and treatment.

## DISCLOSURES

Author contributions: S. Jain: conceptualization, data acquisition, analysis, and interpretation, writing-original draft. A. Dickerson: conceptualization, data acquisition, analysis, and interpretation, writing-original draft. B. Moparty: data acquisition, analysis, and interpretation, supervision, writing-review and editing. H. Kale: data acquisition, analysis, and interpretation, supervision, writing-review & editing. H. Kale is the article guarantor.

Financial disclosure: None to report.

Informed consent was obtained for this case report.
